# Knowledge, attitude, and practice of central line-associated bloodstream infection prevention among intensive care unit nurses in Hebei Province, China: a cross-sectional study

**DOI:** 10.3389/fpubh.2026.1810601

**Published:** 2026-07-01

**Authors:** Jianrong Li, Zhonghua Li, Leping Wang, Xuefang Liu, Junyu Zhu

**Affiliations:** 1 Department of Neurosurgery, The Second Hospital of Hebei Medical University, Shijiazhuang, China; 2 Department of Critical Care Medicine, Luquan Branch, The Second Hospital of Hebei Medical University, Shijiazhuang, China

**Keywords:** catheter-related infections, cross-sectional study, intensive care units, knowledge, attitude, practices, nurses

## Abstract

**Background:**

Central line-associated bloodstream infections (CLABSIs) are a major cause of preventable morbidity and mortality in intensive care units (ICUs).

**Objective:**

This study aimed to assess ICU nurses’ knowledge, attitudes, and practices (KAP) regarding CLABSI prevention and to examine the relationships among the KAP dimensions.

**Methods:**

A cross-sectional study using convenience sampling was conducted in Chinese ICUs from January 6 to March 10, 2025. Registered ICU nurses completed a validated KAP questionnaire. After screening, 471 valid responses were analyzed (81.85% female). Knowledge (9 points), attitude (50 points), and practice (65 points) subscales were summarized using descriptive statistics. Multiple comparison corrections, Spearman correlation analysis, and structural equation modeling (SEM) were used to examine the relationships among KAP dimensions.

**Results:**

Mean (± SD) scores were knowledge 4.03 ± 1.43, attitude 43.19 ± 4.49, and practice 19.97 ± 6.66, indicating insufficient knowledge, generally positive attitudes, and inadequate preventive practices. SEM analysis showed that knowledge had a direct positive effect on attitude (*β* = 0.363, *p* = 0.012) but a direct negative effect on practice (*β* = −0.279, *p* = 0.020); attitude also negatively affected practice (*β* = −0.343, *p* = 0.004). Knowledge further influenced practice indirectly through attitude (*β* = −0.125, *p* = 0.002). Overall, higher knowledge and more positive attitudes were paradoxically associated with lower practice scores.

**Conclusion:**

ICU nurses displayed positive attitudes toward CLABSI prevention but had insufficient knowledge and inadequate preventive practices. The SEM findings suggest that systemic and organizational barriers may hinder the translation of knowledge and attitude into consistent clinical practice. Targeted refresher training and institutional support are needed to improve CLABSI prevention performance.

## Background

Central line-associated bloodstream infections (CLABSIs) are among the most serious healthcare-associated infections in intensive care units (ICUs), contributing substantially to patient morbidity, mortality, and healthcare costs worldwide (1, 2). These infections arise primarily from the frequent use of central venous catheters (CVCs) in critically ill patients (3, 4). In 2019, the pooled mean incidence of CLABSIs in adult ICUs in the United States was 0.8 per 1,000 central line-days (5), while data from the International Nosocomial Infection Control Consortium reported a pooled CLABSI rate of 4.82 per 1,000 line-days across 41 countries (6). A multicenter investigation in China found a mean rate of 1.5 per 1,000 catheter-days, underscoring the continuing need for effective preventive strategies (4). Each episode of CLABSI is associated with significantly greater mortality (up to 39.8% vs. 14.1%) and prolonged ICU stay, incurring additional costs ranging from $3,500 to $65,000 (7–9). Recent international and regional evidence indicates that CLABSI remains a major yet largely preventable challenge in critical care, particularly in settings with uneven healthcare resources and infection prevention capacity (2). Current observational studies and clinical trials primarily focus on physical interventions and device-related complications, such as self-disinfecting connectors or catheter materials. However, the human behavioral factors driving these infections remain underexplored.

Nurses play a critical role in CLABSI prevention because they directly manage catheter insertion sites and ensure compliance with maintenance and infection control protocols (10, 11). Evidence indicates that about 70% of CLABSI events are preventable through adherence to evidence-based practices such as hand hygiene, aseptic technique, regular dressing replacement, and daily assessment of catheter need (9). However, actual adherence to these guidelines varies considerably among ICUs (12).

The Knowledge–Attitude–Practice (KAP) model provides a useful framework for assessing healthcare professionals’ awareness, beliefs, and behaviors related to infection prevention (13–15). However, in ICU settings, the relationships among knowledge, attitudes, and practices may be influenced by workload, institutional support, and other contextual factors, and therefore cannot be assumed to follow a simple linear pattern. Recent applications of SEM in critical care nursing have demonstrated its utility in uncovering complex mediating pathways, such as the role of attitudes in translating knowledge into practice (16). Although previous studies have evaluated ICU nurses’ KAP regarding CLABSI prevention in China and other countries, important gaps remain. Many studies were conducted in single centers or geographically limited settings, and data from Hebei Province are still scarce. In addition, most existing studies have focused on describing KAP levels, whereas fewer have examined the pathways linking these dimensions using analytical approaches such as structural equation modeling. Therefore, region-specific evidence and a deeper understanding of the interrelationships among knowledge, attitudes, and practices are still needed.

Therefore, the present study was conducted in multiple hospitals in Hebei Province, China. The general objective was to assess ICU nurses’ knowledge, attitudes, and practices regarding CLABSI prevention. The specific objectives were to evaluate nurses’ knowledge of CLABSI prevention, examine their attitudes toward CLABSI prevention, assess their preventive practices, and explore factors associated with knowledge, attitude, and practice scores. The findings may help identify modifiable gaps and inform targeted, nurse-centered interventions to strengthen CLABSI prevention.

## Methods

### Study design and participants

This institutional-based cross-sectional study was conducted from January 6 to March 10, 2025, in ICUs of participating hospitals in Hebei Province, China. The participating ICUs were units in which central venous catheters were routinely used and managed for critically ill patients. These ICUs were from hospitals of different levels and types, including public Level 1/2 hospitals, public Level 3 hospitals, and private hospitals. To provide additional structural context for international comparability, information on organizational workload was also collected, including average weekly working hours, nurse-to-patient ratios, and physician-to-nurse ratios at each participating ICU. Registered nurses working in these ICUs were invited to participate using a convenience sampling approach. The source population consisted of registered nurses working in ICUs of participating hospitals in Hebei Province, China, where CVCs were routinely used and managed. The study population comprised eligible ICU nurses who met the inclusion criteria and completed the questionnaire during the study period. The inclusion criteria were: (1) active employment as a registered nurse in an ICU; (2) direct clinical responsibility for the management of patients with CVCs; and (3) provision of written informed consent. The exclusion criteria were: (1) absence from clinical duties (e.g., administrative assignment, leave, or temporary placement in non-CVC units); or (2) work settings in which CVCs were rarely used. Eligible nurses who declined participation or withdrew consent were not enrolled in the study. Refusal to participate was not considered an exclusion criterion, but rather a non-participation decision among otherwise eligible individuals.

Participation in this survey was entirely voluntary and anonymous. The informed consent was obtained from all participants before completing the questionnaire. The study protocol was reviewed and approved by the Institutional Ethics Committee of the Second Hospital of Hebei Medical University (Approval No. 2025-R007), and all procedures were conducted in accordance with the Declaration of Helsinki.

### Procedures

A structured, self-developed questionnaire was used to assess ICU nurses’ KAP regarding CLABSI prevention. The tool was developed following a comprehensive literature review (17) and validated through expert consultation and pilot testing with 42 ICU nurses. Construct validity was subsequently examined by confirmatory factor analysis (CFA), yielding satisfactory fit indices (CMIN/DF = 2.650, RMSEA = 0.059, IFI = 0.862, TLI = 0.848, CFI = 0.861), which confirmed the three-factor structure. The Kaiser-Meyer-Olkin value was 0.892 (*p* < 0.001), indicating sample adequacy. Internal consistency reliability was acceptable, with Cronbach’s *α* = 0.779 in the pilot and 0.786 in the final sample.

### Measures and scoring

The finalized Chinese questionnaire consisted of 42 items across four sections: (1) demographic and professional characteristics (10 items), including age, gender, education, ICU experience, hospital level, and CLABSI-related training; (2) 9 knowledge items scored dichotomously (1 = correct, 0 = incorrect), with a total score ranging from 0 to 9; (3) 10 attitude items rated on a five-point Likert scale from 1 (strongly disagree) to 5 (strongly agree), with a total score ranging from 10 to 50; and (4) 13 practice items rated on a five-point Likert scale from 1 (never) to 5 (always), with a total score ranging from 13 to 65 (18). For interpretation, scores <50% of the total possible score were considered insufficient, scores of 50–75% were considered moderate, and scores >75% were considered adequate.

Convenience sampling was employed in ICUs routinely applying CVCs. The questionnaire was distributed exclusively online via a WeChat-based platform (Questionnaire Star). Participants accessed it through a QR code and completed it anonymously. Anonymization was achieved through automatic removal of IP addresses, timestamps, and any personal identifiers before data export. No personally identifiable information was collected. Each questionnaire response was automatically assigned a random code by the online survey platform, and all data were anonymized prior to analysis. The research team had no access to any information that could identify participants or institutions.

To ensure quality control, each IP address could submit only once, and all items were mandatory. Responses were manually checked for completeness and internal coherence.

In addition to IP restriction, each submitted questionnaire was assigned a unique random code by the online platform, and duplicate or highly similar response patterns were screened during manual data checking. Participants completed the questionnaire independently and anonymously through their own mobile devices. No group discussion or collective completion was organized by the research team. However, because the survey was distributed online within participating ICUs, the possibility that nurses from the same institution may have communicated with each other before or during questionnaire completion could not be completely excluded. Submissions completed in less than 90 s, those exhibiting implausible answer patterns, or those in which all items within any KAP section were answered identically were excluded from the analysis as invalid responses.

### Hospital classification

Participating institutions were categorized according to China’s national three-tier hospital system. Level 1 hospitals provide primary care, Level 2 deliver regional secondary care, and Level 3 are tertiary referral centers with >500 beds and multiple specialties. Levels 1 and 2 were combined in the analysis because of their comparable resource availability, organizational structure, and staffing scale, facilitating a meaningful contrast with Level 3 tertiary facilities and improving comparability for readers from other health systems.

### Sample size estimation

The sample size was estimated based on the commonly used psychometric principle of a 10:1 item-to-respondent ratio for survey studies, which is widely applied in exploratory factor analysis and structural equation modeling. Given the 42-item questionnaire used in this study, a minimum sample size of 420 participants was required. Considering the possibility of invalid responses, additional questionnaires were collected. The final valid sample size was 471, which exceeded the minimum requirement.

### Statistical analysis

Statistical analysis was performed using SPSS 26.0 (IBM Corp., Armonk, NY, USA). Continuous variables were expressed as means ± standard deviations (SD) and categorical variables as frequencies (%). The Kolmogorov–Smirnov test assessed normality. Prior to structural equation modeling, assumptions including multicollinearity were evaluated to ensure data suitability. Normally distributed variables were compared using one-way ANOVA; non-parametric tests were used otherwise. *Post-hoc* Bonferroni adjustments were applied to all pairwise comparisons to control for Type I error arising from multiple testing. Relationships among KAP dimensions were examined by Spearman correlation. While multiple regression analysis is conventionally used to identify independent predictors, SEM was specifically selected for this study because it enables the simultaneous estimation of complex, multi-path relationships—including direct and indirect (mediating) effects-among knowledge, attitude, and practice (19). SEM was performed to explore direct and indirect pathways among knowledge, attitude, and practice, using AMOS 24.0. Model fit was evaluated with root mean square error of approximation (RMSEA), incremental fit index (IFI), Tucker–Lewis index (TLI), and comparative fit index (CFI); thresholds of ≤ 0.08 for RMSEA and ≥ 0.80 for incremental indices indicated satisfactory fit. A two-tailed *p* < 0.05 was considered statistically significant.

## Results

### Participant screening, demographic characteristics, and KAP scores

Initially, 502 questionnaire records were obtained. One eligible nurse declined participation and was excluded. Among the submitted questionnaires, responses with implausible or patterned answers were excluded based on predefined validity screening rules: 4 cases selected all “E” in the knowledge section; 6 cases selected all “B” and 11 cases all “A” in the attitude section; 1 case selected all “A,” 1 case all “C,” and 7 cases all “E” in the practice section. The remaining 471 cases constituted the valid dataset, yielding a response validity rate of 95.98%.

Among valid respondents, 81.85% were female, and the majority (60.51%) were aged 30–40 years. Most worked in public Level 3 hospitals (54.78%) and teaching institutions (96.82%); 94.45% held a bachelor’s degree or higher, and 71.97% had > 5 years of ICU experience. The mean ± SD KAP scores were 4.03 ± 1.43 (possible range 0–9), 43.19 ± 4.49 (possible range 10–50), and 19.97 ± 6.66 (possible range 13–65), respectively.

After multiple-comparison correction, knowledge scores remained significantly associated with education level (Bonferroni-adjusted *p* = 0.018), with bachelor’s-educated nurses scoring higher (4.04 ± 1.42) than those with high school or technical backgrounds (1.67 ± 1.53).

Regarding experience, nurses with > 10 years in the ICU demonstrated higher knowledge scores (4.26 ± 1.43 vs. 3.80 ± 1.44 for 1–5 years; Bonferroni *p* = 0.021). Nurses who received CLABSI-related training scored higher in knowledge (4.12 ± 1.41 vs. 3.56 ± 1.47; Bonferroni *p* = 0.002), whereas those without formal certification (hospital-issued PICC certificates) scored lower (3.05 ± 1.72 vs. 4.13 ± 1.36; Bonferroni *p* < 0.001). The association between strict protocol implementation and lower practice scores (19.01 ± 5.93 vs. 24.19 ± 7.99) remained significant (Bonferroni-adjusted *p* < 0.001).

Comprehensive education on catheter procedures significantly improved attitude (43.94 ± 4.37 vs. 41.82 ± 4.29; Bonferroni *p* < 0.001) and practice (18.96 ± 6.24 vs. 21.82 ± 6.92; Bonferroni *p* < 0.001). Paradoxically, nurses with PICC maintenance experience exhibited lower knowledge scores (3.37 ± 1.58 vs. 4.18 ± 1.35; Bonferroni *p* < 0.001; Table 1; Supplementary Table S1 for post-hoc adjustments).

**Table 1:** Demographic characteristics and KAP scores.

Characteristic	N (%)	Knowledge, mean ± SD	*p*	Attitude, mean ± SD	*p*	Practice, mean ± SD	*p*
Total score		4.03 ± 1.43		43.19 ± 4.49		19.97 ± 6.66	
Gender			0.738		0.056		0.104
Male	85(18.05%)	3.93 ± 1.55		43.94 ± 4.84		19.54 ± 4.49	
Female	386 (81.85%)	4.05 ± 1.41		43.02 ± 4.39		20.07 ± 4.67	
Age, years			0.314		0.054		0.632
20–30	109(23.14%)	3.84 ± 1.38		44.09 ± 4.32		19.63 ± 6.62	
30–40	285(60.51%)	4.06 ± 1.45		42.84 ± 4.58		19.88 ± 6.15	
>40	67(14.23%)	4.14 ± 1.46		43.22 ± 4.25		20.81 ± 8.34	
Education			0.018		0.566		0.184
High school/technical school or below	3(0.64%)	1.67 ± 1.53		44.67 ± 4.93		32.00 ± 18.08	
Bachelor’s degree and above	459(94.45%)	4.04 ± 1.42		43.18 ± 4.49		19.90 ± 6.50	
Years of work experience			0.444		0.796		0.262
<3 years	43(9.13%)	3.67 ± 1.51		43.56 ± 4.29		19.79 ± 5.22	
3–5 years	89(18.90%)	4.10 ± 1.34		42.94 ± 4.61		20.97 ± 7.12	
>5 years	339(71.97%)	4.05 ± 1.44		43.21 ± 4.49		19.74 ± 6.69	
Type of organization			0.163		0.034		0.149
Public Level 1/2 Hospital	204(43.31%)	3.91 ± 1.49		42.61 ± 4.59		20.53 ± 6.90	
Public Level 3 Hospital	258(54.78%)	4.14 ± 1.37		43.62 ± 4.36		19.60 ± 6.52	
Private Hospital	9(1.91%)	3.33 ± 1.66		43.78 ± 4.55		18.11 ± 3.89	
Teaching hospital			0.010		0.361		0.824
Yes	456(96.82%)	4.06 ± 1.42		43.16 ± 4.47		19.93 ± 6.55	
No	15(3.18%)	3.07 ± 1.49		44.07 ± 5.05		21.27 ± 9.72	
Years working in the ICU			0.021		0.663		0.663
1–5 years	191(40.55%)	3.80 ± 1.44		43.41 ± 4.35		20.24 ± 7.02	
5–10 years	121(25.69%)	4.07 ± 1.38		43.30 ± 3.97		19.73 ± 5.65	
> 10 years	159(33.76%)	4.26 ± 1.43		42.84 ± 5.00		19.85 ± 6.94	
Current position			0.315		0.640		0.450
Intern nurse	8(1.70%)	3.13 ± 1.89		45.13 ± 5.77		19.25 ± 5.50	
Registered nurse	174(36.94%)	3.90 ± 1.46		43.00 ± 4.58		20.65 ± 7.04	
Charge nurse	252(53.50%)	4.15 ± 1.39		43.25 ± 4.39		19.48 ± 6.26	
Deputy head nurse	15(3.18%)	3.87 ± 1.51		42.27 ± 4.91		20.27 ± 5.87	
Head nurse	22(4.67%)	4.00 ± 1.38		43.91 ± 4.08		20.41 ± 8.66	
Number of beds in workplace			0.507		0.166		0.454
5–10	85(18.05%)	3.84 ± 1.47		42.69 ± 4.37		20.86 ± 7.99	
10–20	204(43.31%)	4.10 ± 1.38		43.10 ± 4.19		20.12 ± 6.46	
>20	182(38.64%)	4.03 ± 1.47		43.52 ± 4.84		19.40 ± 6.16	
Internal care protocol for CLABSI			0.568		0.045		0.026
Yes	444(94.27%)	4.03 ± 1.41		43.28 ± 4.49		19.83 ± 6.57	
No	27(5.73%)	3.89 ± 1.76		41.74 ± 4.27		22.37 ± 7.69	
Training related to CLABSI			0.002		0.773		0.382
Yes	391(83.01%)	4.12 ± 1.41		43.19 ± 4.56		19.78 ± 6.30	
No	80(16.99%)	3.56 ± 1.47		43.18 ± 4.16		20.91 ± 8.18	
Studied the best practice guidelines for the prevention of CLABSI			0.099		0.030		0.004
Yes	388(82.38%)	4.08 ± 1.40		43.39 ± 4.52		19.56 ± 6.24	
No	83(17.62%)	3.78 ± 1.58		42.25 ± 4.21		21.90 ± 8.10	
Need to learn more about CLABSI			0.030		0.506		0.411
Yes	452(95.97%)	4.06 ± 1.43		43.16 ± 4.48		19.99 ± 6.57	
No	19(4.03%)	3.32 ± 1.34		43.84 ± 4.79		19.58 ± 8.73	
PICC insertion certification			0.075		0.170		0.672
Yes	87(18.47%)	3.74 ± 1.47		43.79 ± 4.83		20.08 ± 7.36	
No	384(81.53%)	4.09 ± 1.42		43.05 ± 4.40		19.95 ± 6.50	
Type of PICC certification			<0.001		0.127		0.447
Hospital-issued certificate	40(8.49%)	3.05 ± 1.72		44.58 ± 5.07		21.08 ± 9.87	
Certificate issued by a professional society	45(9.55%)	3.98 ± 1.44		43.09 ± 4.88		22.09 ± 9.50	
No certificate	386(81.95%)	4.13 ± 1.36		43.06 ± 4.36		19.61 ± 5.76	
PICC maintenance experience			<0.001		0.326		0.161
Yes	90(19.11%)	3.37 ± 1.58		42.61 ± 5.37		21.93 ± 9.48	
No	381(80.89%)	4.18 ± 1.35		43.33 ± 4.25		19.51 ± 5.72	
Hospital with SOPs for the prevention of CLABSI			0.020		0.191		0.263
Yes	455(96.60%)	4.06 ± 1.41		43.15 ± 4.46		19.82 ± 6.34	
No	16(3.40%)	3.13 ± 1.78		44.38 ± 5.28		24.25 ± 12.35	
Hospital have a CLABSI prevention protocol that is strictly implemented			0.053		0.098		<0.001
Yes, and it is strictly implemented	377(80.04%)	4.02 ± 1.42		43.41 ± 4.50		19.01 ± 5.93	
Yes, but implementation needs improvement	89(18.90%)	4.12 ± 1.48		42.25 ± 4.31		24.19 ± 7.99	
No	5(1.06%)	2.80 ± 0.45		43.00 ± 4.95		18.00 ± 3.94	
Education on the standard procedures for central venous catheter insertion and maintenance			0.044		<0.001		<0.001
Yes	308(65.39%)	4.13 ± 1.47		43.94 ± 4.37		18.96 ± 6.24	
A little, but not comprehensive	154(32.70%)	3.84 ± 1.32		41.82 ± 4.29		21.82 ± 6.92	
Never	9(1.91%)	3.56 ± 1.59		40.89 ± 5.84		23.11 ± 8.78	
Education related to CLABSI prevention			0.075		<0.001		<0.001
Yes	315(66.88%)	4.12 ± 1.40		43.86 ± 4.39		18.98 ± 6.19	
A little, but not comprehensive	144(30.57%)	3.85 ± 1.47		41.92 ± 4.17		21.99 ± 7.16	
Never	12(2.55%)	3.50 ± 1.73		40.75 ± 6.58		21.92 ± 6.93	

ICU, Intensive care unit; CLABSI, Central line-associated bloodstream infection; PICC, Peripherally Inserted Central Catheter; SOPs, Standard Operating Procedures.

### Distribution of knowledge, attitude, and practice dimension

Only 2.34% correctly recognized that the CVC connector should be changed weekly (K8). Similarly, 7.43% knew that CVCs require maintenance every 3 days (K5), and 33.76% correctly identified that the disinfection radius should be 15 cm (K6; Table 2). In the attitude dimension, 27.39% disagreed and 6.37% strongly disagreed that the CVC should be promptly removed when a patient develops a fever (A7); 15.92% disagreed and 1.06% strongly disagreed that CVCs may cause serious infectious complications (A8); 5.31% disagreed and 1.70% strongly disagreed that palpation helps identify infection (A3; Table 3). Regarding practice, 10.83% rarely and 31.00% never bathe patients with CVCs in chlorhexidine solution (P3); 3.40% rarely and 3.40% never avoid the puncture site when disinfecting (P8); 4.03% rarely and 2.34% never inspect the skin or dressing daily (P1; Table 4).

**Table 2:** Distribution of responses across knowledge dimension items (*N* = 471).

Items	A, n (%)	B, n (%)	C, n (%)	D, n (%)	E, n (%)
1. CLABSI refers to an infection that occurs during central venous catheter (CVC) placement or within how long after catheter removal?	25(5.31%)	6(1.27%)	150(31.85%)	279(59.24%)	11(2.34%)
2. In patients with renal failure, which puncture site should be avoided?	49(10.40%)	289(61.36%)	27(5.73%)	29(6.16%)	77(16.35%)
3. If signs of CLABSI are present, the catheter should be removed and the tip cut aseptically and placed in a sterile test tube for immediate bacterial culture. How many centimeters from the tip should be taken?	92(19.53%)	90(19.11%)	36(7.64%)	229(48.62%)	24(5.10%)
4. If a CVC is placed emergently without ensuring sterile technique, the catheter must be replaced immediately and should not exceed how many hours in place?	74(15.71%)	263(55.84%)	6(1.27%)	105(22.29%)	23(4.88%)
5. Under normal circumstances, how often should a CVC be maintained?	9(1.91%)	35(7.43%)	29(6.16%)	387(82.17%)	11(2.34%)
6. When changing a dressing film, what is the disinfection radius from the center of the puncture site?	12(2.55%)	78(16.56%)	159(33.76%)	218(46.28%)	4(0.85%)
7. During continuous infusion, how often should the infusion set be replaced?	15(3.18%)	435(92.36%)	12(2.55%)	7(1.49%)	2(0.42%)
8. How often should the connector of a CVC be replaced?	198(42.04%)	18(3.82%)	56(11.89%)	188(39.92%)	11(2.34%)
9. When CLABSI is suspected in a patient, which of the following findings can confirm the diagnosis of CLABSI?	121(25.69%)	100(21.23%)	33(7.01%)	196(41.61%)	21(4.46%)

CLABSI, Central line associated bloodstream infection; CVC, central venous catheter.

**Table 3:** Distribution of attitude dimension responses.

Items	Strongly agree, n (%)	Agree, n (%)	Uncertain, n (%)	Disagree, n (%)	Strongly disagree, n (%)
1. I am very interested in knowledge about CLABSI.	224(47.56%)	216(45.86%)	29(6.16%)	1(0.21%)	1(0.21%)
2. I believe that nurses with in-depth knowledge of catheter-related infections can reduce the incidence of CLABSI.	322(68.37%)	136(28.87%)	11(2.34%)	1(0.21%)	1(0.21%)
3. I believe that palpation at the catheter site helps identify signs of infection.	202(42.89%)	165(35.03%)	71(15.07%)	25(5.31%)	8(1.70%)
4. I believe that preventing CLABSI is very important for the treatment and prognosis of a patient’s illness.	314(66.67%)	147(31.21%)	10(2.12%)	0(0.00%)	0(0.00%)
5. I believe hand hygiene must be performed before catheter insertion and dressing changes.	390(82.80%)	76(16.14%)	4(0.85%)	1(0.21%)	0(0.00%)
6. I believe that regularly replacing CVCs is an effective measure for preventing CLABSI.	274(58.17%)	117(24.84%)	43(9.13%)	30(6.37%)	7(1.49%)
7. I believe that the CVC should be promptly removed when the patient develops a fever.	106(22.51%)	66(14.01%)	140(29.72%)	129(27.39%)	30(6.37%)
8. I believe that CVCs are devices that may lead to serious infectious complications.	167(35.46%)	145(30.79%)	79(16.77%)	75(15.92%)	5(1.06%)
9. I believe nurses play a critical role in the prevention of CLABSI.	313(66.45%)	142(30.15%)	15(3.18%)	1(0.21%)	0(0.00%)
10. I believe nurses should assess the necessity of catheter retention daily and promptly remove any unnecessary catheters.	304(64.54%)	144(30.57%)	18(3.82%)	3(0.64%)	2(0.42%)

CLABSI, Central line associated bloodstream infection; CVC, central venous catheter.

**Table 4:** Distribution of responses for practice dimension items (*N* = 471).

Items	Never, n (%)	Occasionally, n (%)	Sometimes, n (%)	Often, n (%)	Always, n (%)
1. I assess the skin condition around the catheter insertion site and the dressing status daily to determine whether the patient is at risk for bloodstream infection.	11(2.34%)	19(4.03%)	23(4.88%)	152(32.27%)	266(56.48%)
2. If the patient reports pain at the catheter site, I will remove the dressing to conduct a thorough examination.	9(1.91%)	29(6.16%)	59(12.53%)	155(32.91%)	219(46.50%)
3. I regularly bathe patients with a CVC using a solution containing chlorhexidine.	146(31.00%)	51(10.83%)	69(14.65%)	94(19.96%)	111(23.57%)
4. When infusing fluids or replacing a CVC, I vigorously scrub the needleless connector, catheter hub, and threaded cross-section.	8(1.70%)	14(2.97%)	17(3.61%)	105(22.29%)	327(69.43%)
5. Before administering medication through a CVC, I aspirate blood and confirm catheter placement depth.	4(0.85%)	10(2.12%)	16(3.40%)	94(19.96%)	347(73.67%)
6. When infusing fluids or replacing a needleless connector, I disinfect with the insertion site as the center, ensuring a disinfection area with a diameter ≥ 20 cm and a duration ≥ 15 s.	5(1.06%)	11(2.34%)	15(3.18%)	100(21.23%)	340(72.19%)
7. Before inserting a test tube, administering medication via CVC, or after removing an old dressing, I strictly follow hand hygiene procedures for no less than 15 s.	2(0.42%)	10(2.12%)	6(1.27%)	97(20.59%)	356(75.58%)
8. I avoid the puncture site when disinfecting with an alcohol swab.	16(3.40%)	16(3.40%)	20(4.25%)	86(18.26%)	333(70.70%)
9. When inspecting or palpating a CVC, I perform hand hygiene and wear sterile gloves.	3(0.64%)	11(2.34%)	14(2.97%)	95(20.17%)	348(73.89%)
10. If I notice bleeding at the wound, contamination (or suspected contamination) of the dressing, moisture, detachment, loosening, or anything that may endanger the catheter, I immediately replace the dressing and ask the patient about their condition.	1(0.21%)	6(1.27%)	6(1.27%)	93(19.75%)	365(77.49%)

CVC, central venous catheter.

### Correlation analysis

Spearman analysis identified a significant negative correlation between attitude and practice (*r* = −0.461, *p* < 0.001). No statistically significant correlation was observed between knowledge and either attitude or practice (Table 5).

**Table 5:** Spearman rank correlation coefficients among knowledge, attitude, and practice scores.

Variable	Knowledge	Attitude	Practice
Knowledge	1		
Attitude	−0.008 (*p* = 0.866)	1	
Practice	−0.081 (*p* = 0.080)	−0.461 (*p* < 0.001)	1

### Structural equation model analysis

**Figure 1:**
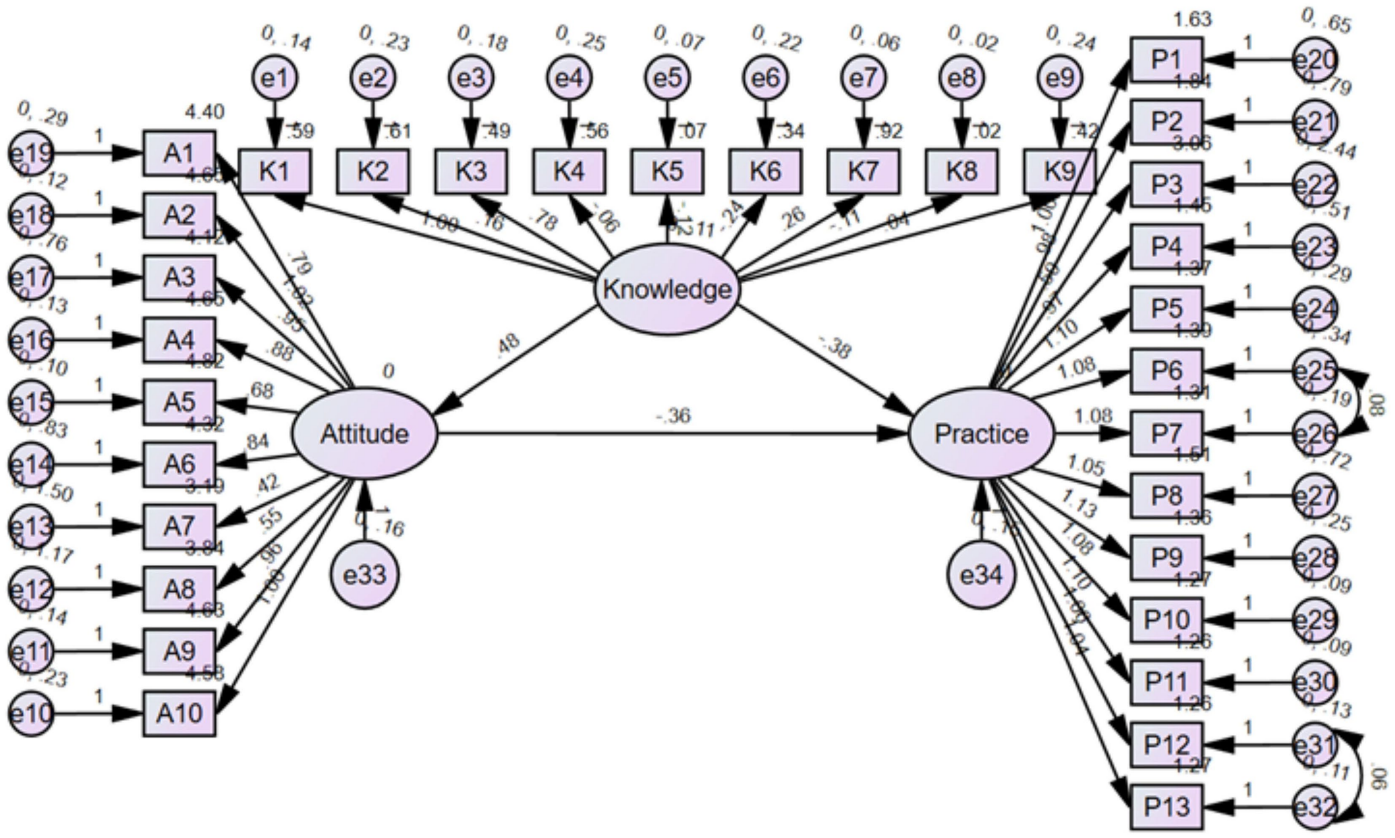
Structural equation model showing direct and indirect pathways among knowledge, attitude, and practice regarding central line–associated bloodstream infection (CLABSI) prevention. Standardized path coefficients (*β*) and *p* values are indicated.

The SEM model exhibited adequate fit (CMIN/DF = 3.232, RMSEA = 0.069, IFI = 0.808, TLI = 0.791, CFI = 0.806; Supplementary Table S2). Knowledge had a direct positive effect on attitude (*β* = 0.363, *p* = 0.012) and a direct negative effect on practice (*β* = −0.279, *p* = 0.020). Attitude exerted a negative effect on practice (*β* = −0.343, *p* = 0.004). Additionally, knowledge indirectly affected practice through attitude (*β* = −0.125, *p* = 0.002; Supplementary Table S3; Figure 1).

## Discussion

A previous cross-sectional study among ICU nurses also reported insufficient knowledge and generally positive attitudes toward CLABSI prevention. This is consistent with the overall pattern observed in the present study. However, unlike earlier studies that reported more favorable associations between knowledge, attitudes, and preventive behavior, our structural equation model showed negative direct associations between knowledge, attitude, and practice. This discrepancy may reflect contextual differences in workload, institutional support, and the feasibility of implementing recommended preventive measures in daily ICU practice (20, 21).

An unexpected finding was that nurses with peripherally inserted central catheter (PICC) experience scored lower in knowledge. This may reflect overreliance on routine practice or outdated experience rather than current standards. Inadequate standardization of PICC training could also contribute, suggesting that certification alone cannot guarantee contemporary knowledge. Prior research similarly reported decreases in evidence-based knowledge among individuals with longer clinical experience (22, 23), reinforcing the need for ongoing professional renewal rather than one-time certification.

The SEM revealed complex relationships among the KAP dimensions: knowledge positively influenced attitude, but both knowledge and attitude negatively influenced practice. This finding suggests that knowledge and favorable attitudes may not translate directly into preventive behavior when ICU nurses face contextual barriers such as workload, limited resources, or inconsistent protocol implementation. Clinically, this indicates that CLABSI prevention should not rely only on knowledge training, but should also strengthen workflow support, supervision, and feasible implementation of standardized protocols (21).

The negative direct effects of knowledge and attitude on practice present a particularly counterintuitive phenomenon that necessitates a more profound theoretical exploration. While traditional KAP models imply a positive progression, our findings resonate with recent findings highlighting implementation barriers, high workload, and psychological stress in intensive care environments, particularly in low- and middle-income countries (24, 25). Importantly, this counterintuitive phenomenon is not isolated. Recent SEM-based KAP studies among ICU nurses and healthcare professionals have similarly reported direct negative effects of knowledge on clinical practice, underscoring a pervasive disconnect between cognition and behavior in high-pressure medical settings (26, 27). Within Chinese ICUs, nurses frequently encounter high patient-to-nurse ratios, demanding workloads, and staffing challenges that directly impact infection control compliance (28, 29). In such contexts, a robust awareness of optimal practices (knowledge) coupled with a strong conviction in their significance (attitude) can paradoxically lead to increased frustration, moral distress, or a sense of futility when these best practices are perceived as unattainable given existing structural constraints. This situation may precipitate cognitive dissonance or a perceived lack of control, subsequently resulting in a withdrawal from ideal behaviors and perpetuating a negative association (30). Furthermore, the adverse influence of attitude on practice may stem from the realization that, despite a strong commitment to preventing CLABSI, systemic barriers obstruct consistent action based on those positive attitudes. It is also plausible that certain questionnaire items, particularly those pertinent to practice, did not adequately encompass these pervasive institutional barriers, leading to suppressor effects wherein the true relationship is obscured by unmeasured confounding factors such as institutional safety culture and economic constraints (31). This complexity underscores that enhancing CLABSI prevention in this context necessitates more than mere nurse education; it demands fundamental changes in the enabling environment, as corroborated by Mohammadi and Fooladi (2022), who identify organizational factors as critical contributors to the knowledge-practice gap (32).

Analysis of item-level responses reinforced this interpretation. Nurses adhered more consistently to routine, low-burden tasks, but less to time-intensive measures such as chlorhexidine bathing or daily skin assessment. This pattern is consistent with observations from other studies showing that task complexity, workload, and feedback strongly influence adherence to infection-control behaviors and subsequent economic burden (33, 34). Consequently, interventions must address structural feasibility, not only cognitive knowledge. This assertion is supported by Das and Choudhury (2019), who demonstrated that hand hygiene compliance diminishes without continuous reinforcement, thus underscoring the need for sustained educational initiatives (35).

These findings identify modifiable gaps and can inform targeted, nurse-centered interventions to strengthen CLABSI prevention in intensive care units within Hebei Province. Given regional differences in healthcare systems and resources, the results should be interpreted as context-specific rather than generalized to other provinces or countries. Future nationwide multicenter studies are warranted to validate and extend these observations. First, at the structural and organizational level, it is imperative to critically evaluate ICU workflows, nurse staffing levels, and resource availability. The implementation of standardized, visible, and consistently reinforced CLABSI prevention protocols that are feasible within existing constraints essential, and enhancing the visibility of such policies in nursing literature and institutional guidelines is crucial for fostering nurse involvement in policymaking and compliance (36). This may require the incorporation of CLABSI indicators into routine quality monitoring through digitized, electronic information management systems to ensure timely and accurate data tracking, and the cultivation of a robust organizational culture that prioritizes infection prevention while ensuring consistent feedback and accountability (37). Second, targeted educational interventions should extend beyond mere theoretical knowledge transfer. Programs must emphasize context-specific scenarios, simulation-based training, skill repetition, and critical decision-making in uncertain situations, addressing the identified specific knowledge gaps (e.g., CVC connector changes, disinfection techniques) (38, 39). Third, in light of the concerning findings regarding nurses with PICC experience, a comprehensive review of existing certification and ongoing professional development programs is warranted to ensure they are current, evidence-based, and effectively translate into safe practice (40, 41). These implications are corroborated by Almutairi and Almutairi (2021), who found that educational interventions enhance knowledge and compliance, particularly among experienced nurses (5). These findings challenge the linear assumptions of traditional KAP models, which posit that knowledge fosters positive attitudes and, in turn, leads to better practices. In contrast, the structural equation model in this study revealed negative direct effects of both knowledge and attitudes on practice, indicating a more complex, context-dependent relationship. This suggests that the KAP framework may require adaptation when applied in high-acuity environments like ICUs, where systemic and psychological barriers may distort the expected progression from knowledge to action. Thus, the current study contributes to refining the KAP model by emphasizing the need to incorporate environmental and emotional variables into its theoretical structure.

Given regional context and healthcare system differences, these findings should not be generalized to other countries. European and North American institutions typically maintain lower CLABSI rates due to stricter structural prerequisites and long-standing infection-control frameworks (42). Nevertheless, the present study contributes region-specific evidence that can guide targeted interventions in Chinese ICUs, where contextual constraints differ. Its scientific value lies in providing empirical data for localized quality-improvement strategies, not in claiming universal applicability.

### Strengths

This study has several strengths. First, it focused on ICU nurses, who play a direct role in CVC maintenance and CLABSI prevention, making the findings clinically relevant to infection control practice. Second, the study included 471 valid responses from ICUs of participating hospitals in Hebei Province, providing region-specific evidence on nurses’ KAP regarding CLABSI prevention. Third, the questionnaire was developed based on literature review, expert consultation, and pilot testing, and its reliability and construct validity were evaluated before formal analysis. Finally, in addition to describing knowledge, attitude, and practice levels, this study used SEM to explore the direct and indirect pathways among the KAP dimensions, which provided a more detailed understanding of their interrelationships.

### Limitations

This study has several limitations. First, the cross-sectional design precludes inference of causality among knowledge, attitudes, and practices. Second, the data were collected using an online self-reported questionnaire, which may have introduced social desirability bias, recall bias, and response validity concerns, although anonymity was used to reduce these risks. Third, convenience sampling rather than random sampling was used, which may have introduced selection bias. In addition, the sample had a strong representation of tertiary and teaching hospitals, limiting the generalizability of the findings to smaller institutions or other settings. Finally, nurses’ practices were assessed by self-report rather than direct observational assessment; therefore, the reported practice scores may not fully reflect actual bedside behavior. Furthermore, because the questionnaire was distributed online within participating ICUs, selection bias related to online access and potential response influence among nurses from the same institution could not be fully excluded.

## Conclusion

This study showed that ICU nurses in Hebei Province had insufficient knowledge, generally positive attitudes, and suboptimal practices regarding CLABSI prevention. The SEM results indicated that higher knowledge and more positive attitudes were paradoxically associated with lower practice scores, suggesting that knowledge alone may not be sufficient to improve preventive behavior in ICU settings. Based on these findings, hospitals should strengthen regular training on key CLABSI prevention procedures, improve implementation and supervision of standardized protocols, and provide greater organizational support to facilitate evidence-based practice. Further multicenter studies are needed to confirm these findings in other settings.

## Data Availability

The original contributions presented in the study are included in the article/Supplementary material, further inquiries can be directed to the corresponding author.
