# Supplementary material for: Knowledge, attitude, and practice of central line-associated bloodstream infection prevention among intensive care unit nurses in Hebei Province, China: a cross-sectional study
Source: Front Public Health. 2026 Jul 1;14:1810601. doi: 10.3389/fpubh.2026.1810601 (PMC13368873; doi:10.3389/fpubh.2026.1810601)
Supplement: Supplementary file 1 [file Supplementary_file_1.DOC]

**Supplementary table 1.** Relationships between Participant Characteristics and Knowledge, Attitude, and Practice Scores

| **Variable** | **Comparison** | **Knowledge** | | | **Attitude** | | | **Practice** | | |
| --- | --- | --- | --- | --- | --- | --- | --- | --- | --- | --- |
|  |  | Test Statistic | *P* | Bonferroni *P* | Test Statistic | *P* | Bonferroni *P* | Test Statistic | *P* | Bonferroni *P* |
| **Age** |  |  |  |  |  |  |  |  |  |  |
|  | 20–30 vs. 30–40 years | –28.974 | 0.055 | 0.164 | 44.09 ± 4.32 |  |  | 19.63 ± 6.62 |  |  |
|  | 20–30 vs. ≥40 years | –54.687 | 0.006 | 0.018 | 42.84 ± 4.58 |  |  | 19.88 ± 6.15 |  |  |
|  | 30–40 vs. ≥40 years | –25.713 | 0.135 | 0.405 | 43.22 ± 4.25 |  |  | 20.81 ± 8.34 |  |  |
| **Years of Work Experience** |  |  |  |  |  |  |  |  |  |  |
|  | ≤3  vs. 3–5 | –48.774 | 0.050 | 0.150 | 43.56 ± 4.29 |  |  | 19.79 ± 5.22 |  |  |
|  | ≤3  vs. >5 | –86.245 | <0.001 | <0.001 | 42.94 ± 4.61 |  |  | 20.97 ± 7.12 |  |  |
|  | 3–5  vs. >5 | –37.471 | 0.019 | 0.056 | 43.21 ± 4.49 |  |  | 19.74 ± 6.69 |  |  |
| **Type of Institution** |  |  |  |  |  |  |  |  |  |  |
|  | Public Level 1/2 vs. Private Hospital | –46.211 | 0.311 | 0.933 | –32.781 | 0.010 | 0.030 |  |  |  |
|  | Public Level 1/2 vs. Public Level 3 Hospital | –55.366 | <0.001 | <0.001 | –32.806 | 0.478 | >0.999 |  |  |  |
|  | Public Level 3 vs. Private Hospital | 9.155 | 0.840 | >0.999 | –0.025 | >0.999 | >0.999 |  |  |  |
| **Years Working in ICU** |  |  |  |  |  |  |  |  |  |  |
|  | 1–5 vs. 5–10 | –29.732 | 0.056 | 0.168 | 43.41 ± 4.35 |  |  | 20.24 ± 7.02 |  |  |
|  | 1–5 vs. >10 | –61.457 | <0.001 | <0.001 | 43.30 ± 3.97 |  |  | 19.73 ± 5.65 |  |  |
|  | 5–10 vs. >10 | –31.725 | 0.050 | 0.149 | 42.84 ± 5.00 |  |  | 19.85 ± 6.94 |  |  |
| **Position/Title** |  |  |  |  |  |  |  |  |  |  |
|  | Intern Nurse vs. Registered Nurse | –142.213 | 0.003 | 0.033 |  |  |  |  |  |  |
|  | Intern Nurse vs. Head Nurse | –148.341 | 0.007 | 0.073 |  |  |  |  |  |  |
|  | Intern Nurse vs. Deputy Head Nurse | –171.000 | 0.004 | 0.035 |  |  |  |  |  |  |
|  | Intern Nurse vs. Charge Nurse | –196.415 | <0.001 | <0.001 |  |  |  |  |  |  |
|  | Registered Nurse vs. Head Nurse | –6.128 | 0.840 | >0.999 |  |  |  |  |  |  |
|  | Registered Nurse vs. Deputy Head Nurse | –28.787 | 0.424 | >0.999 |  |  |  |  |  |  |
|  | Registered Nurse vs. Charge Nurse | –54.202 | <0.001 | <0.001 |  |  |  |  |  |  |
|  | Head Nurse vs. Deputy Head Nurse | 22.659 | 0.613 | >0.999 |  |  |  |  |  |  |
|  | Head Nurse vs. Charge Nurse | 48.074 | 0.106 | >0.999 |  |  |  |  |  |  |
|  | Deputy Head Nurse vs. Charge Nurse | 25.415 | 0.475 | >0.999 |  |  |  |  |  |  |
| **Type of Catheter Certification** |  |  |  |  |  |  |  |  |  |  |
|  | Hospital Certificate vs. Society Certificate | –80.195 | <0.001 | 0.001 |  |  |  |  |  |  |
|  | Hospital Certificate vs. No Certificate | –101.682 | <0.001 | 0.001 |  |  |  |  |  |  |
|  | Society Certificate vs. No Certificate | 21.487 | 0.308 | 0.925 |  |  |  |  |  |  |
| **CLABSI Prevention Protocol in Hospital** |  |  |  |  |  |  |  |  |  |  |
|  | Strictly Implemented vs. Needs Improvement |  |  |  |  |  |  | 11.647 | 0.849 | >0.009 |
|  | Strictly Implemented vs. None |  |  |  |  |  |  | 120.011 | 0.054 | 0.163 |
|  | Needs Improvement vs. None |  |  |  |  |  |  | –108.364 | <0.001 | <0.001 |
| **Training on Central Venous Catheterization and Maintenance** |  |  |  |  |  |  |  |  |  |  |
|  | Trained vs. Partially Trained | 35.623 | 0.007 | 0.021 | 66.716 | <0.001 | <0.001 | –72.943 | <0.001 | <0.001 |
|  | Trained vs. Never Trained | 69.758 | 0.123 | 0.370 | 73.722 | 0.108 | 0.325 | –78.120 | 0.089 | 0.266 |
|  | Partially Trained vs. Never Trained | 34.135 | 0.457 | >0.999 | 7.006 | 0.880 | >0.999 | –5.177 | 0.911 | >0.999 |
| **Training on CLABSI Prevention Knowledge** |  |  |  |  |  |  |  |  |  |  |
|  | Trained vs. Partially Trained | 45.442 | <0.001 | 0.002 | 58.895 | <0.001 | <0.001 | –73.730 | <0.001 | <0.001 |
|  | Trained vs. Never Trained | 54.105 | 0.170 | 0.509 | 72.093 | 0.071 | 0.213 | –66.400 | 0.096 | 0.288 |
|  | Partially Trained vs. Never Trained | 8.663 | 0.830 | >0.999 | 13.198 | 0.746 | >0.999 | 7.330 | 0.857 | >0.999 |

**Supplementary table 2. SEM fit indicators.**

| Model fitting indicators | Ref. | Measured results |
| --- | --- | --- |
| CMIN/DF | 1-3 excellent，3-5 good | 3.232 |
| RMSEA | <0.08 good | 0.069 |
| IFI | >0.8 good | 0.808 |
| TLI | >0.8 good | 0.791 |
| CFI | >0.8 good | 0.806 |

**Supplementary table 3. Analysis of direct and indirect effects.**

| Model paths | Standardized direct effects (95%CI) | P | Standardized indirect effects (95%CI) | P |
| --- | --- | --- | --- | --- |
| Knowledge→Attitude | 0.363 (0.194-0.526) | 0.012 |  |  |
| Knowledge→Practice | -0.279 (-0.483- -0.061) | 0.020 | -0.125 (-0.243- -0.073) | 0.002 |
| Attitude→Practice | -0.343 (-0.526- -0.213) | 0.004 |  |  |
