# Supplementary material for: Knowledge, attitude, and practice of central line-associated bloodstream infection prevention among intensive care unit nurses in Hebei Province, China: a cross-sectional study
Source: Front Public Health. 2026 Jul 1;14:1810601. doi: 10.3389/fpubh.2026.1810601 (PMC13368873; doi:10.3389/fpubh.2026.1810601)
Supplement: Supplementary file 2 [file Supplementary_file_2.DOCX]

Questionnaire on ICU Nurses' Knowledge and Attitudes Regarding Central Line-Associated Bloodstream Infections (CLABSI-KAP-Q)

Hello! We are conducting a study on the *knowledge, attitudes, and practices of ICU nurses regarding the prevention of central line-associated bloodstream infections (CLABSI)*. You are invited to participate in this research by completing the following questionnaire. This questionnaire consists of four parts: Part 1: Basic Information (Demographics) – 10 items. Part 2: Knowledge about CLABSI – 9 items. Part 3: Attitudes toward CLABSI – 10 items. Part 4: CLABSI-related Practices – 13 items. It will take approximately 20 minutes to complete. Please fill it out carefully. Your responses will remain anonymous and confidential and will not negatively impact your work or life.
Thank you for your support!

Part 1: Basic Information

Gender

🞎Male 🞎Female

Age

How many years have you worked in the ICU?

What is your current position?
🞎Intern Nurse

🞎Registered Nurse

🞎 Charge Nurse

🞎 Deputy Head Nurse

🞎 Head Nurse

What is your highest level of education?
🞎Associate Degree

🞎Bachelor’s Degree

🞎Master’s Degree

🞎Above Master’s

How many beds are there in your workplace?

Does your hospital have an internal care protocol or standard for CLABSI?

🞎Yes 🞎No

Have you ever attended any training related to CLABSI?
🞎Yes 🞎No

Are you familiar with or have you studied the best practice guidelines for the prevention and control of CLABSI?
🞎Yes 🞎No

Do you think you need to learn more about CLABSI?

🞎Yes 🞎No

Part 2: Knowledge about CLABSI

1.CLABSI refers to an infection that occurs during central venous catheter (CVC) placement or within how long after catheter removal?
A. 8 hours
B. 16 hours
C. 24 hours
D. 48 hours
E. Not sure

2.In patients with renal failure, which puncture site should be avoided?
A. Subclavian vein
B. Femoral vein
C. Internal jugular vein
D. External jugular vein
E. Not sure

3. If signs of CLABSI are present, the catheter should be removed and the tip cut aseptically and placed in a sterile test tube for immediate bacterial culture. How many centimeters from the tip should be taken?
A. 2 cm
B. 3 cm
C. 4 cm
D. 5 cm
E. Not sure

4. If a CVC is placed emergently without ensuring sterile technique, the catheter must be replaced immediately and should not exceed how many hours in place?
A. 12 hours
B. 24 hours
C. 36 hours
D. 48 hours
E. Not sure

5. Under normal circumstances, how often should a CVC be maintained?
A. Once a day
B. Every 3 days
C. Every 5 days
D. Once a week
E. Not sure

6.When changing a dressing film, what is the disinfection radius from the center of the puncture site?
A. 5 cm
B. 10 cm
C. 15 cm
D. 20 cm
E. Not sure

7. During continuous infusion, how often should the infusion set be replaced?
A. Once every 12 hours
B. Once every 24 hours
C. Once every 48 hours
D. Replaced irregularly
E. Not sure

8. How often should the connector of a CVC be replaced?
A. Every 24 hours
B. Every 48 hours
C. Every 72 hours
D. Once a week
E. Not sure

9.When CLABSI is suspected in a patient, which of the following findings can confirm the diagnosis of CLABSI?
A. Peripheral blood culture is negative for bacteria or fungi, but the catheter tip culture is positive.
B. The same pathogen is cultured from both the catheter tip and peripheral blood.
C. Different pathogens with the same susceptibility profile are cultured from the catheter tip and peripheral blood.
D. The same pathogen with identical susceptibility results is cultured from both the catheter tip and peripheral blood.
E. Not sure

Part 3: Attitudes Toward CLABSI

1. I am very interested in knowledge about CLABSI.
🞎 Strongly agree 🞎 Agree 🞎 Uncertain 🞎 Disagree 🞎 Strongly disagree

2. I believe that nurses with in-depth knowledge of catheter-related infections can reduce the incidence of CLABSI.
🞎 Strongly agree 🞎 Agree 🞎 Uncertain 🞎 Disagree 🞎 Strongly disagree

3. I believe that palpation at the catheter site helps identify signs of infection.

🞎 Strongly agree 🞎 Agree 🞎 Uncertain 🞎 Disagree 🞎 Strongly disagree

4. I believe that preventing CLABSI is very important for the treatment and prognosis of a patient's illness.

🞎 Strongly agree 🞎 Agree 🞎 Uncertain 🞎 Disagree 🞎 Strongly disagree

5. I believe hand hygiene must be performed before catheter insertion and dressing changes.

🞎 Strongly agree 🞎 Agree 🞎 Uncertain 🞎 Disagree 🞎 Strongly disagree

6. I believe that regularly replacing CVCs is an effective measure for preventing CLABSI.

🞎 Strongly agree 🞎 Agree 🞎 Uncertain 🞎 Disagree 🞎 Strongly disagree

7. I believe that the CVC should be promptly removed when the patient develops a fever.

🞎 Strongly agree 🞎 Agree 🞎 Uncertain 🞎 Disagree 🞎 Strongly disagree

8. I believe that CVCs are devices that may lead to serious infectious complications.

🞎 Strongly agree 🞎 Agree 🞎 Uncertain 🞎 Disagree 🞎 Strongly disagree

9. I believe nurses play a critical role in the prevention of CLABSI.

🞎 Strongly agree 🞎 Agree 🞎 Uncertain 🞎 Disagree 🞎 Strongly disagree

10. I believe nurses should assess the necessity of catheter retention daily and promptly remove any unnecessary catheters.

🞎 Strongly agree 🞎 Agree 🞎 Uncertain 🞎 Disagree 🞎 Strongly disagree

Part 4: Practices Related to CLABSI

1. I assess the skin condition around the catheter insertion site and the dressing status daily to determine whether the patient is at risk for bloodstream infection.

🞎Never 🞎Occasionally 🞎Sometimes 🞎Often 🞎Always

2. If the patient reports pain at the catheter site, I will remove the dressing to conduct a thorough examination.

🞎Never 🞎Occasionally 🞎Sometimes 🞎Often 🞎Always

3. I regularly bathe patients with a CVC using a solution containing chlorhexidine.

🞎Never 🞎Occasionally 🞎Sometimes 🞎Often 🞎Always

4. When infusing fluids or replacing a CVC, I vigorously scrub the needleless connector, catheter hub, and threaded cross-section.

🞎Never 🞎Occasionally 🞎Sometimes 🞎Often 🞎Always

5. Before administering medication through a CVC, I aspirate blood and confirm catheter placement depth.

🞎Never 🞎Occasionally 🞎Sometimes 🞎Often 🞎Always

6. When infusing fluids or replacing a needleless connector, I disinfect with the insertion site as the center, ensuring a disinfection area with a diameter ≥ 20 cm and a duration ≥ 15 seconds.

🞎Never 🞎Occasionally 🞎Sometimes 🞎Often 🞎Always

7. Before inserting a test tube, administering medication via CVC, or after removing an old dressing, I strictly follow hand hygiene procedures for no less than 15 seconds.

🞎Never 🞎Occasionally 🞎Sometimes 🞎Often 🞎Always

8. I avoid the puncture site when disinfecting with an alcohol swab.

🞎Never 🞎Occasionally 🞎Sometimes 🞎Often 🞎Always

9. When inspecting or palpating a CVC, I perform hand hygiene and wear sterile gloves.

🞎Never 🞎Occasionally 🞎Sometimes 🞎Often 🞎Always

10. If I notice bleeding at the wound, contamination (or suspected contamination) of the dressing, moisture, detachment, loosening, or anything that may endanger the catheter, I immediately replace the dressing and ask the patient about their condition.

🞎Never 🞎Occasionally 🞎Sometimes 🞎Often 🞎Always

11. If I observe fever or other signs of infection in the patient, I promptly report it to the physician.

🞎Never 🞎Occasionally 🞎Sometimes 🞎Often 🞎Always

12. During shift handover, I report the condition of the patient’s catheter to the next on-duty nurse.

🞎Never 🞎Occasionally 🞎Sometimes 🞎Often 🞎Always

13. During ward rounds, I check catheter patency, ask the patient about their condition, and address issues promptly.

🞎Never 🞎Occasionally 🞎Sometimes 🞎Often 🞎Always

Copyright: Xing Wuran, Li Huiju, Fang Pingdang, March 18, 2022
Reprinted with permission: Wu Ran, Li Huiju, Fang Pingdang, February 21, 2023
